# Supplementary material for: Variation in the Local Grey Mullet Populations (Mugil cephalus) on the Western Pacific Fringe
Source: Genes (Basel). 2024 Sep 29;15(10):1280. doi: 10.3390/genes15101280 (PMC11508091; doi:10.3390/genes15101280)
Supplement: Supplementary file 1 [file genes-15-01280-s001.zip › genes-3196866-supplementary.pdf]

Table S1. Genotype frequencies and Nei's (1977) *F*-statistics of loci for all *Mugil cephalus* sampled. Localities in bold type indicate nonmigrating local populations while those in normal type indicate migrating populations.

Locus	**Tapong (542)**	**Kaoping (36)**	**Peimen (73)**	**Tadu (42)**	**Tanshui (158)**	**Dashi (77)**	**Hualien (23)**	Kaoshiung (131)	Chiding (20)	Anping (31)	Wuchi (25)	Matsu (56)	Tachen (9)	Shanghai (22)	Nagasaki (41)	F_{IS}	F_{IT}	F_{ST}
mAAT	-100/-100 (542)	-100/-100 (36)	-100/-100 (71)	-100/-100 (42)	-100/-100 (158)	-100/-100 (77)	-100/-100 (23)	-100/-100 (127)	-100/-100 (20)	-100/-100 (31)	-100/-100 (25)	-100/-100 (53)	-100/-100 (9)	-100/-100 (21)	-100/-100 (41)	0.524	0.536	0.026
	-20/-20 (2)													-20/-20 (1)				
			-127/127 (1)															
	-100/-127 (1)		-100/-127 (1)					-100/-127 (1)				-100/-127 (3)						
	-100/-20 (1)							-100/-20 (3)										
H_o	0.004	0.000	0.014	0.000	0.000	0.000	0.000	0.031	0.000	0.000	0.000	0.054	0.000	0.000	0.000			
CK-A	100/100 (519)	100/100 (44)	100/100 (70)	100/100 (42)	100/100 (151)	100/100 (73)	100/100 (23)	100/100 (131)	100/100 (20)	100/100 (31)	100/100 (25)	100/100 (54)	100/100 (8)	100/100 (22)	100/100 (37)	0.028	0.049	0.021
	100/119 (22)	100/119 (1)	100/119 (3)		100/119 (7)	100/119 (3)						100/119 (2)	100/119 (1)		100/119 (4)			
	119/119 (1)					119/119 (1)												
H_o	0.041	0.028	0.041	0.000	0.044	0.039	0.000	0.000	0.000	0.000	0.000	0.036	0.111	0.000	0.098			
GPI-A	100/100 (86)		100/100 (6)	100/100 (13)	100/100 (64)	100/100 (33)	100/100 (7)	100/100 (130)	100/100 (19)	100/100 (31)	100/100 (24)	100/100 (47)	100/100 (9)	100/100 (21)	100/100 (7)	0.352	0.669	0.489***
	135/135 (332)	135/135 (29)	135/135 (43)	135/135 (19)	135/135 (53)	135/135 (26)	135/135 (8)					135/135 (3)			***130/130 (19)***			
	100/117 (4)				100/117 (1)			100/117 (1)						100/117 (1)				
	100/135 (86)	100/135 (4)	100/135 (21)	100/135 (10)	100/135 (37)	100/135 (18)	100/135 (8)				100/135 (1)	100/135 (5)			***100/130 (14)***			
	117/135 (31)	117/135 (3)	117/135 (3)		117/135 (2)										***117/130 (1)***			
	100/75 (3)				100/75 (1)				100/75 (1)			100/75 (1)						
H_o	0.229	0.194	0.329	0.238	0.259	0.234	0.348	0.008	0.050	0.000	0.040	0.107	0.000	0.045	0.366			

continued

Locus	**Tapong (542)**	**Kaoping (36)**	**Peimen (73)**	**Tadu (42)**	**Tanshui (158)**	**Dashi (77)**	**Hualien (23)**	Kaoshiung (131)	Chiding (20)	Anping (31)	Wuchi (25)	Matsu (56)	Tachen (9)	Shanghai (22)	Nagasaki (41)	F_{IS}	F_{IT}	F_{ST}
GPI-B	**100/100 (522)**	**100/100 (36)**	**100/100 (71)**	**100/100 (40)**	**100/100 (151)**	**100/100 (73)**	**100/100 (22)**	100/100 (132)	100/100 (20)	100/100 (31)	100/100 (25)	100/100 (56)	100/100 (9)	100/100 (22)	100/100 (39)	-0.021	0.010	0.011
	**100/140 (19)**		**100/140 (2)**	**100/140 (2)**	**100/140 (6)**	**100/140 (3)**	**100/140 (1)**	100/140 (1)							100/140 (2)			
	**100/20 (1)**				**100/20 (1)**	**100/20 (1)**												
H_o	**0.036**	**0.000**	**0.027**	**0.048**	**0.044**	**0.052**	**0.043**	0.008	0.000	0.000	0.000	0.000	0.000	0.000	0.049			
IDH-A	**100/100 (541)**	**100/100 (36)**	**100/100 (73)**	**100/100 (42)**	**100/100 (158)**	**100/100 (77)**	**100/100 (23)**	100/100 (131)	100/100 (20)	100/100 (31)	100/100 (25)	100/100 (55)	100/100 (9)	100/100 (22)	100/100 (41)	-0.008	-0.001	0.007
	**100/123 (1)**											100/123 (1)						
H_o	**0.002**	**0.000**	**0.000**	**0.000**	**0.000**	**0.000**	**0.000**	0.000	0.000	0.000	0.000	0.018	0.000	0.000	0.000			
IDH-B	**100/100 (542)**	**100/100 (36)**	**100/100 (73)**	**100/100 (42)**	**100/100 (157)**	**100/100 (76)**	**100/100 (23)**	100/100 (129)	100/100 (20)	100/100 (29)	100/100 (25)	100/100 (55)	100/100 (9)	100/100 (21)	100/100 (41)	-0.022	-0.005	0.017
					**100/120 (1)**	**100/120 (1)**		100/120 (2)		100/120 (2)		100/120 (1)		100/120 (1)				
H_o	**0.000**	**0.000**	**0.000**	**0.000**	**0.006**	**0.013**	**0.000**	0.015	0.000	0.065	0.000	0.018	0.000	0.045	0.000			
LDH-A	**100/100 (541)**	**100/100 (36)**	**100/100 (73)**	**100/100 (42)**	**100/100 (158)**	**100/100 (77)**	**100/100 (23)**	100/100 (131)	100/100 (20)	100/100 (31)	100/100 (23)	100/100 (55)	100/100 (9)	100/100 (21)	100/100 (41)	-0.034	-0.004	0.029
	**100/125 (1)**										100/225 (2)			100/225 (1)				
H_o	**0.002**	**0.000**	**0.000**	**0.000**	**0.000**	**0.000**	**0.000**	0.000	0.000	0.000	0.040	0.000	0.000	0.045	0.000			
LDH-B	**100/100 (529)**	**100/100 (36)**	**100/100 (73)**	**100/100 (42)**	**100/100 (157)**	**100/100 (77)**	**100/100 (23)**	100/100 (131)	100/100 (20)	100/100 (30)	100/100 (25)	100/100 (56)	100/100 (8)	100/100 (21)	100/100 (41)	-0.038	-0.007	0.030
	**100/136 (13)**				**100/136 (1)**					100/136 (1)			100/136 (1)	100/136 (1)				
H_o	**0.024**	**0.000**	**0.000**	**0.000**	**0.006**	**0.000**	**0.000**	0.000	0.000	0.032	0.000	0.000	0.111	0.045	0.000			

continued

Locus	**Tapong (542)**	**Kaoping (36)**	**Peimen (73)**	**Tadu (42)**	**Tanshui (158)**	**Dashi (77)**	**Hualien (23)**	Kaoshiung (131)	Chiding (20)	Anping (31)	Wuchi (25)	Matsu (56)	Tachen (9)	Shanghai (22)	Nagasaki (41)	F_{IS}	F_{IT}	F_{ST}
MDH-A	**100/100 (540)**	**100/100 (36)**	**100/100 (73)**	**100/100 (42)**	**100/100 (157)**	**100/100 (77)**	**100/100 (23)**	100/100 (131)	100/100 (20)	100/100 (31)	100/100 (25)	100/100 (56)	100/100 (9)	100/100 (22)	100/100 (41)	-0.003	0.000	0.002
	**100/67 (1)**				**100/67 (1)**													
H_o	**0.004**	**0.000**	**0.000**	**0.000**	**0.006**	**0.000**	**0.000**	0.000	0.000	0.000	0.000	0.000	0.000	0.000	0.000			
MPI	**100/100 (540)**	**100/100 (36)**	**100/100 (71)**	**100/100 (40)**	**100/100 (151)**	**100/100 (75)**	**100/100 (23)**	100/100 (131)	100/100 (20)	100/100 (31)	100/100 (25)	100/100 (55)	100/100 (8)	100/100 (18)	100/100 (40)	0.059	0.089	0.032
	**100/112 (2)**		**100/112 (2)**	**100/112 (2)**	**100/112 (7)**	**100/112 (2)**						100/112 (1)		100/112 (1)	100/112 (1)			
	**100/90 (1)**												100/90 (1)	100/90 (3)				
	**90/90 (1)**																	
H_o	**0.002**	**0.000**	**0.027**	**0.048**	**0.044**	**0.026**	**0.000**	0.000	0.000	0.000	0.000	0.021	0.111	0.182	0.000			
PGM-A	**100/100 (540)**	**100/100 (36)**	**100/100 (71)**	**100/100 (42)**	**100/100 (155)**	**100/100 (77)**	**100/100 (22)**	100/100 (131)	100/100 (20)	100/100 (31)	100/100 (25)	100/100 (56)	100/100 (9)	100/100 (22)	100/100 (41)	-0.016	-0.003	0.013
	**100/130 (1)**																	
	**100/50 (1)**		**100/50 (2)**		**100/50 (3)**		**100/50 (1)**											
H_o	**0.004**	**0.000**	**0.027**	**0.000**	**0.019**	**0.000**	**0.043**	0.000	0.000	0.000	0.000	0.000	0.000	0.000	0.000			
PGM-B	**100/100 (541)**	**100/100 (36)**	**100/100 (73)**	**100/100 (42)**	**100/100 (156)**	**100/100 (77)**	**100/100 (23)**	100/100 (131)	100/100 (20)	100/100 (31)	100/100 (25)	100/100 (56)	100/100 (9)	100/100 (22)	100/100 (41)	0.604	0.687	0.008
	**100/125 (1)**				**125/125 (1)**													
					**100/125 (1)**													
H_o	**0.002**	**0.000**	**0.000**	**0.000**	**0.006**	**0.000**	**0.000**	0.000	0.000	0.000	0.000	0.000	0.000	0.000	0.000			

continued

Locus	**Tapong (540)**	**Kaoping (36)**	**Peimen (73)**	**Tadu (42)**	**Tanshui (158)**	**Dashi (77)**	**Hualien (23)**	Kaoshiung (131)	Chiding (20)	Anping (31)	Wuchi (25)	Matzu (56)	Tachen (9)	Shanghai (22)	Nagasaki (41)	F_{IS}	F_{IT}	F_{ST}
PGDH	**100/100 (295)**	**100/100 (13)**	**100/100 (51)**	**100/100 (40)**	**100/100 (141)**	**100/100 (70)**	**100/100 (21)**	100/100 (127)	100/100 (20)	100/100 (27)	100/100 (23)	100/100 (55)	100/100 (8)	100/100 (20)	100/100 (38)	0.332	0.460	0.202**
	**125/125 (113)**	**125/125 (12)**	**125/125 (8)**		**125/125 (8)**	**125/125 (1)**	**125/125 (1)**							125/125 (1)				
	**100/125 (131)**	**100/125 (11)**	**100/125 (14)**	**100/125 (2)**	**125/100 (12)**	**125/100 (4)**	**100/125 (1)**	100/125 (3)		100/125 (4)			100/100 (1)	100/125 (1)	100/125 (1)			
	**100/80 (3)**				**100/80 (1)**	**125/100 (2)**		100/80 (1)			100/80 (2)	100/80 (1)			100/80 (2)			
H_o	**0.247**	**0.306**	**0.192**	**0.048**	**0.057**	**0.078**	**0.043**	0.031	0.000	0.129	0.080	0.018	0.111	0.045	0.073			
Mean																0.270	0.521	0.344***
P_{95}	**15.4**	**15.4**	**15.4**	**7.7**	**15.4**	**15.48**	**15.4**	0.0	0.0	7.7	0	7.7	30.8	7.7	7.7			
N_a (±SE)	**2.5 (±0.2)**	**1.3 (±0.2)**	**1.6 (±0.2)**	**1.3 (±0.1)**	**2.1 (±0.2)**	**1.6 (±0.2)**	**1.3 (±0.1)**	1.5 (±0.2)	1.1 (±0.1)	1.2 (±0.1)	1.2 (±0.1)	1.6 (±0.2)	1.3 (±0.1)	1.6 (±0.2)	1.5 (±0.2)			
H_o (±SE)	**0.046 (±0.024)**	**0.041 (±0.027)**	**0.051 (±0.027)**	**0.029 (±0.018)**	**0.038 (±0.019)**	**0.034 (±0.018)**	**0.037 (±0.026)**	0.007 (±0.003)	0.004 (±0.004)	0.017 (±0.011)	0.015 (±0.009)	0.021 (±0.009)	0.034 (±0.015)	0.031 (±0.014)	0.045 (±0.028)			
H_e (±SE)	**0.076 (±0.044)**	**0.055 (±0.040)**	**0.049 (±0.038)**	**0.049 (±0.038)**	**0.065 (±0.039)**	**0.058 (±0.038)**	**0.056 (±0.039)**	0.007 (±0.003)	0.004 (±0.004)	0.017 (±0.010)	0.015 (±0.008)	0.027 (±0.015)	0.034 (±0.015)	0.038 (±0.014)	0.056 (±0.036)			

Table S2. Genotype frequencies of *Mugil cephalus* juveniles, with some metabolic allozymes.

Locus	Tanshui（621）	Fulung（426）	Fangliao（423）	F_{IT}	F_{IT}	F_{ST}
GPI-A	100/100（183）	100/100（146）	100/100（57）	0.3134	03272	0.0201
	135/135（268）	135/135（215）	135/135（242）			
	100/117（1）					
	100/135（158）	100/135（57）	100/135（106）			
	117/135（6）	117/135（8）	117/135（17）			
	100/75（3）					
	135/75（2）		135/75（1）			
Ho	0.4998	0.437	0.4155			
GPI-B	100/100（566）	100/100（371）	100/100（385）	-0.0353	-0.0325	0.0027
	100/140（45）	100/140（52）	100/140（31）			
	100/20（9）	100/20（2）	100/20（7）			
	100/200（1）					
		140/140（1）				
Ho	0.0852	0.1231	0.0864			
MPI	100/100（614）	100/100（421）	100/100（421）	-0.0041	-0.0037	0.0004
	100/112（2）	100/112（1）				
	100/90（4）	100/90（4）	100/90（2）			
	100/50（1）					
Ho	0.0112	0.0117	0.0047			
PGM-A	100/100（615）	100/100（424）	100/100（419）	-0.0036	-0.0027	0.0008
	100/50（5）		100/50（1)			
	100/75（1）	100/75（1）				
		100/130（1）	100/130（3）			
Ho	0.0096	0.0047	0.0094			
PGM-B	100/100（620）	100/100（425）	100/100（423）	-0.001	-0.0007	0.0004
	100/125（1）	100/125（1）				
Ho	0.0016	0.0023	0.0000			
P_{95}	20%	40%	20%			
H_O（±SE）	0.0770 ± 0.1155	0.14014+0.1545	0.0794+0.1784			
H_E（±SE）	0.8784+0.2143	0.8841+0.1868	0.8967+0.1784			
N_a	3.4+0.89	2.8+0.45	2.6+1.14			

Table S3. Matrix of pairwise *F*st (below diagonal) and R_{ST} (above diagonal) among 15 populations based on allozyme loci in *Mugil cephalus*. Bold type letters indicate statistically significant results.

	NA	SH	TC	MS	DS	TS	WC	TD	PM	AP	CD	KS	KP	TP	HL	FLJ	TSJ	FLJ
NA		**0.285**	**0.410**	**0.437**	0.003	0.003	**0.300**	-0.005	**0.087**	**0.480**	**0.491**	**0.537**	**0.219**	**0.082**	0.007	0.001	-0.003	**0.044**
SH	**0.360**		-0.030	0.002	**0.216**	**0.239**	-0.009	**0.260**	**0.441**	0.007	-0.001	**0.032**	**0.456**	**0.398**	**0.182**	**0.328**	**0.302**	**0.458**
TC	**0.366**	-0.006		-0.007	**0.290**	**0.257**	-0.027	**0.340**	**0.527**	0.025	0.031	-0.001	**0.580**	**0.414**	**0.289**	**0.336**	**0.304**	**0.474**
MS	**0.469**	0.029	0.016		**0.326**	**0.275**	0.026	**0.392**	**0.558**	**0.028**	-0.002	**0.021**	**0.621**	**0.423**	**0.332**	**0.344**	**0.310**	**0.479**
DS	**0.308**	**0.242**	**0.246**	**0.309**		-0.004	**0.229**	0.004	**0.129**	**0.351**	**0.357**	**0.425**	**0.236**	**0.108**	-0.013	0.017	0.007	**0.092**
TS	**0.315**	**0.251**	**0.252**	**0.265**	-0.003		**0.248**	0.004	**0.101**	**0.281**	**0.289**	**0.333**	**0.194**	**0.096**	-0.008	**0.016**	**0.008**	**0.080**
WC	**0.421**	0.022	0.008	0.002	**0.277**	**0.253**		**0.273**	**0.458**	0.032	0.014	**0.056**	**0.488**	**0.412**	**0.201**	**0.328**	**0.302**	**0.458**
TD	**0.341**	**0.324**	**0.343**	**0.420**	0.004	0.003	**0.381**		**0.067**	**0.413**	**0.413**	**0.503**	**0.180**	**0.074**	**0.005**	0.004	-0.003	**0.036**
PM	**0.395**	**0.433**	**0.448**	**0.510**	**0.111**	**0.095**	**0.473**	**0.068**		**0.569**	**0.577**	**0.639**	**0.063**	0.012	**0.135**	**0.068**	**0.071**	**0.026**
AP	**0.428**	0.007	0.025	0.022	**0.294**	**0.268**	0.019	**0.395**	**0.480**		0.028	-0.004	**0.632**	**0.417**	**0.371**	**0.353**	**0.320**	**0.489**
CD	**0.426**	0.029	0.031	-0.010	**0.287**	**0.258**	0.006	**0.397**	**0.482**	0.027		-0.005	**0.644**	**0.434**	**0.383**	**0.360**	**0.327**	**0.496**
KS	**0.546**	0.022	0.032	0.015	**0.392**	**0.324**	0.019	**0.497**	**0.585**	0.001	0.015		**0.705**	**0.452**	**0.440**	**0.383**	**0.341**	**0.518**
KP	**0.456**	**0.490**	**0.514**	**0.608**	**0.265**	**0.249**	**0.557**	**0.245**	**0.096**	**0.551**	**0.569**	**0.676**		0.015	**0.236**	**0.188**	**0.185**	**0.161**
TP	**0.355**	**0.371**	**0.384**	**0.388**	**0.125**	**0.113**	**0.379**	**0.102**	**0.019**	**0.379**	**0.386**	**0.419**	0.023		**0.108**	**0.088**	**0.092**	**0.067**
HL	**0.311**	**0.242**	**0.266**	**0.350**	-0.010	-0.008	**0.308**	0.005	**0.115**	**0.318**	**0.333**	**0.419**	**0.261**	**0.121**		0.017	0.007	**0.100**
FLJ	**0.373**	**0.350**	**0.348**	**0.338**	0.014	0.018	**0.343**	-0.003	**0.088**	**0.363**	**0.347**	**0.393**	**0.294**	**0.132**	**0.021**		0.001	**0.033**
TSJ	**0.379**	**0.347**	**0.345**	**0.347**	0.021	0.013	**0.337**	-0.001	**0.098**	**0.357**	**0.340**	**0.378**	**0.309**	**0.142**	**0.014**	0.001		**0.038**
LBJ	**0.468**	**0.502**	**0.507**	**0.505**	**0.106**	**0.087**	**0.502**	**0.042**	**0.054**	**0.518**	**0.506**	**0.545**	**0.299**	**0.117**	**0.116**	**0.041**	**0.034**	
